# Thermodynamic evidence of magnetic-field-induced complete valley polarization in bismuth

**DOI:** 10.1038/s41598-018-38206-x

**Published:** 2019-02-08

**Authors:** Ayumu Iwasa, Akihiro Kondo, Shiro Kawachi, Kazuto Akiba, Yoshiki Nakanishi, Masahito Yoshizawa, Masashi Tokunaga, Koichi Kindo

**Affiliations:** 1 Institute for Solid State Physics (ISSP), The University of Tokyo, Kashiwa, Chiba 277–8581 Japan; 2 Graduate School of Arts and Science, Iwate University, Morioka, Iwate 020-8551 Japan

## Abstract

We investigated the fundamental physical properties in the ultra-quantum limit state of bismuth through measurements of magnetoresistance, magnetization, magnetostriction, and ultrasound attenuation in magnetic fields up to 60T. For magnetic fields applied along the bisectrix direction of a single crystal, a drastic sign reversal in magnetostriction was observed at approximately 39T, which could be ascribed to the complete valley polarization in the electron Fermi pockets. The application of magnetic fields along the binary direction presented an anomalous feature at approximately 50T only in the magnetoresistance. The emergence of a field-induced splitting of a valley was proposed as a possible origin of this anomaly.

## Introduction

Some materials have multiple constant energy surfaces [or Fermi surfaces (FSs)] at different positions in the reciprocal space, which are referred to as valleys. In addition to the electron and spin degrees of freedom, the valley degree of freedom has recently attracted great attention, not only for its fundamental scientific importance but also for its potential application to “valleytronics”, which exploits the valley degree of freedom to carry the quantum information^1–5^. The relative carrier densities in the valleys, or the valley polarization, can usually be controlled through the optical pumping method^2,4^. The valley polarized state realized in the excited state, however, disappears rapidly in a few nanoseconds^3^. Static valley polarization can be caused by the application of a magnetic field^6–11^. In particular, Zhu *et al*. recently inferred that bismuth undergoes complete valley polarization in high magnetic fields^10,11^. Since various fundamental phenomena of metals were first discovered in elemental bismuth^12^, unambiguous evaluation of the static valley polarization in this material is essentially important to clarify the canonical behaviour.

**Figure 1:**
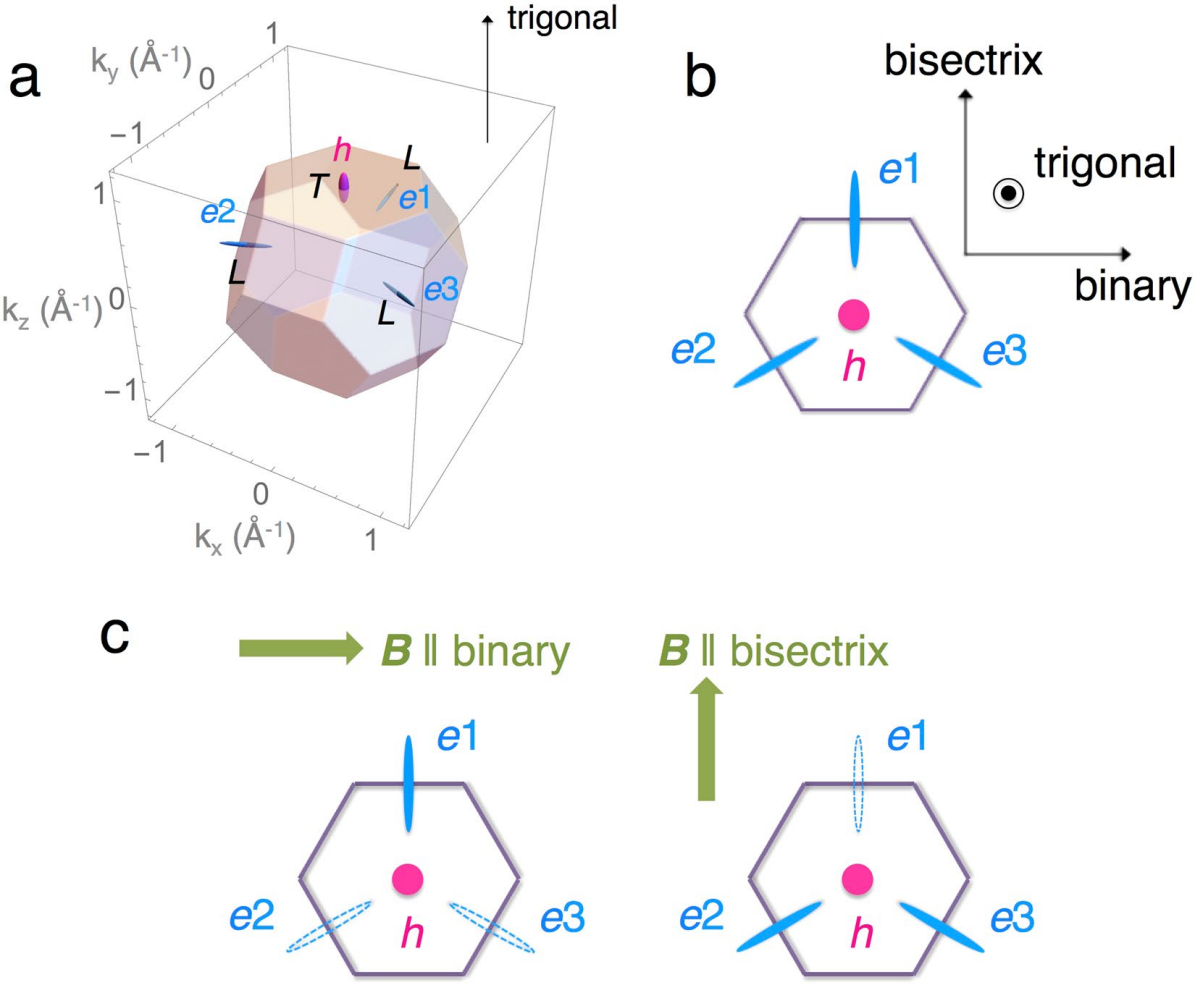
(**a**) Three-dimensional schematics of the first Brillouin zone and Fermi surfaces (FSs) of bismuth. Three valleys of electron pockets denoted by *e*1, *e*2, and *e*3 (light blue) locate at the *L*-points, and one hole-pocket valley denoted by *h* (magenta) locates at the *T*-point. (**b**) Schematic of the FSs projected onto the binary-bisectrix plane. (**c**) Schematic of the field-induced static valley polarization of the electron pockets for *B* || binary and *B* || bisectrix proposed by the quadratic model. Depopulated pockets are represented as open ellipsoids.

Bismuth has a nearly equal number of electrons and holes with densities of 10^17^ cm^−3^. The FSs consist of one hole pocket at the *T*-point and three electron pockets at the *L*-points in the first Brillouin zone of the rhombohedral cell. These three equivalent electron pockets are considered as three-fold degenerate electron valleys (*e*1, *e*2, *e*3) in zero magnetic field (Fig. 1a,b). Owing to the highly anisotropic shape of the electron pockets as illustrated in Fig. 1a,b, the three-fold valley degeneracy can be lifted in a rotatable magnetic field^6,10,11,13^. At the *L*-points, bismuth has narrow band gaps (*E*_g_ ~ 15.3 meV) between the conduction and the valence bands. Owing to the strong spin-orbit interaction (*λ* ~ 1.8 eV), electrons at the *L*-point exhibit an almost linear dispersion relation and can be regarded as massive Dirac fermions^14,15^. Application of a magnetic field (*B*) along both the binary and bisectrix axes is considered first to reduce the gap at the *L*-points^10,11,16^ and then cause avoided level-crossing at *B*_c_^17,18^. With further increasing *B*, the hole and electron subbands will move in the opposite directions and finally cause a semimetal–semiconductor transition at a certain field^16,19^.

Recently, Zhu *et al*. carried out angle-resolved magnetotransport measurements in bismuth up to 65T and deduced the underlying energy spectra of the Landau levels (LLs)^10^. Their results suggested that complete valley polarization could be selectively achieved depending on the direction of the applied field: only one of the three electron valleys (*e*1) is occupied in magnetic fields *B* > 57T || binary, while two of the valleys (*e*2 and *e*3) are occupied in *B* > 39T || bisectrix (Fig. 1c). This interpretation is based on the model that takes into account quadratic *B* dependence in the Zeeman energy for the lowest LL, which we refer as the quadratic model in the following^11,18,20^. This scenario, however, contradicts with the early interpretation that predicts the *e*1 valley completely depopulates first at *B* ~ 18T || binary^16^. This early model takes only *B* linear term in the Zeeman energy into account (linear model)^16^. The difference between these two models becomes clear in the field range beyond 50T, and therefore, the accurate determination of the LL spectrum in this field range is crucially important to identify the actual style of valley polarization caused by magnetic fields.

In this report, we address the complete valley polarization in single crystals of bismuth through magnetotransport, magnetization, magnetostriction, and ultrasonic attenuation measurements in pulsed high magnetic fields up to 60T. Among these physical quantities, magnetostriction is especially sensitive to the filling of the electronic states, and hence, can provide us with the direct thermodynamic evidence of valley polarization in the ultra-quantum limit state as shown later.

## Results

**Figure 2:**
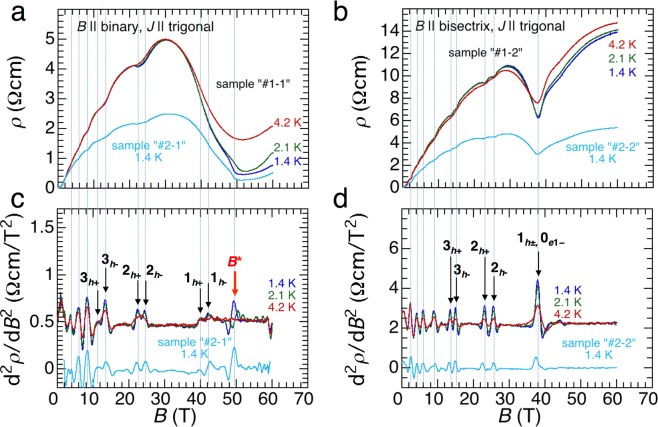
Transverse magnetoresistance (MR) of bismuth single crystals in magnetic fields up to 60T for (**a**) *B* || binary and (**b**) *B* || bisectrix. Electrical currents were applied along the trigonal axis for both experiments. (**c**,**d**) Second derivatives of the MR for *B* || binary and *B* || bisectrix, respectively. In (**c**,**d)**, results for different sample pieces were shifted vertically for clarity. The dotted vertical lines represent the peak fields in the d^2^*ρ/*d*B*^2^ curves. The thin black arrows and labels denote the Landau indices assigned by the quadratic models, and the thick red arrow at B^*^ denotes the anomaly that cannot be assigned in both linear and quadratic models.

Figure 2 shows the transverse magnetoresistance (MR) measured at temperatures of 1.4, 2.1, and 4.2 K (for sample ‘#1-1’) in magnetic fields applied along the binary axis (Fig. 2a) and bisectrix axis (Fig. 2b). In both cases, electric currents were applied along the trigonal axis. We measured two different sample pieces ‘#1-1’ and ‘#2-1’ for *B* || binary and ‘#1-2’ and ‘#2-2’ for *B* || bisectrix. All these samples were taken from the same crystalline bismuth ingot. In agreement with the previous measurements^10,16,21^, all the samples exhibit clear Shubnikov–de Haas (SdH) oscillations. In the case of compensated semimetals such as bismuth, local minima in the transverse MR correspond to the field in which the LL crosses the Fermi energy (*E*_F_). The local minima in the MR appear as local maxima in the second derivative of the MR, as indicated by solid black arrows in Fig. 2c,d. The indices shown in Fig. 2c,d were assigned based on the quadratic model. Here, subscripts ± represent spin splitting in each subband. The Zeeman splitting in the hole LLs may be caused by the finite misalignment of the field, which has also been observed in earlier studies^10,16,21^. The linear model results in almost the same assignment for *B* || binary up to 60T. Here, the emergence of the peak structure at approximately 50T for *B* || binary (B^*^) cannot be explained in the both models (a thick red arrow in Fig. 2c). As seen in Fig. 2c, this peak structure moves to higher fields and is quickly damped with increasing temperature, contrary to the other peak structures originating from SdH oscillations.

**Figure 3:**
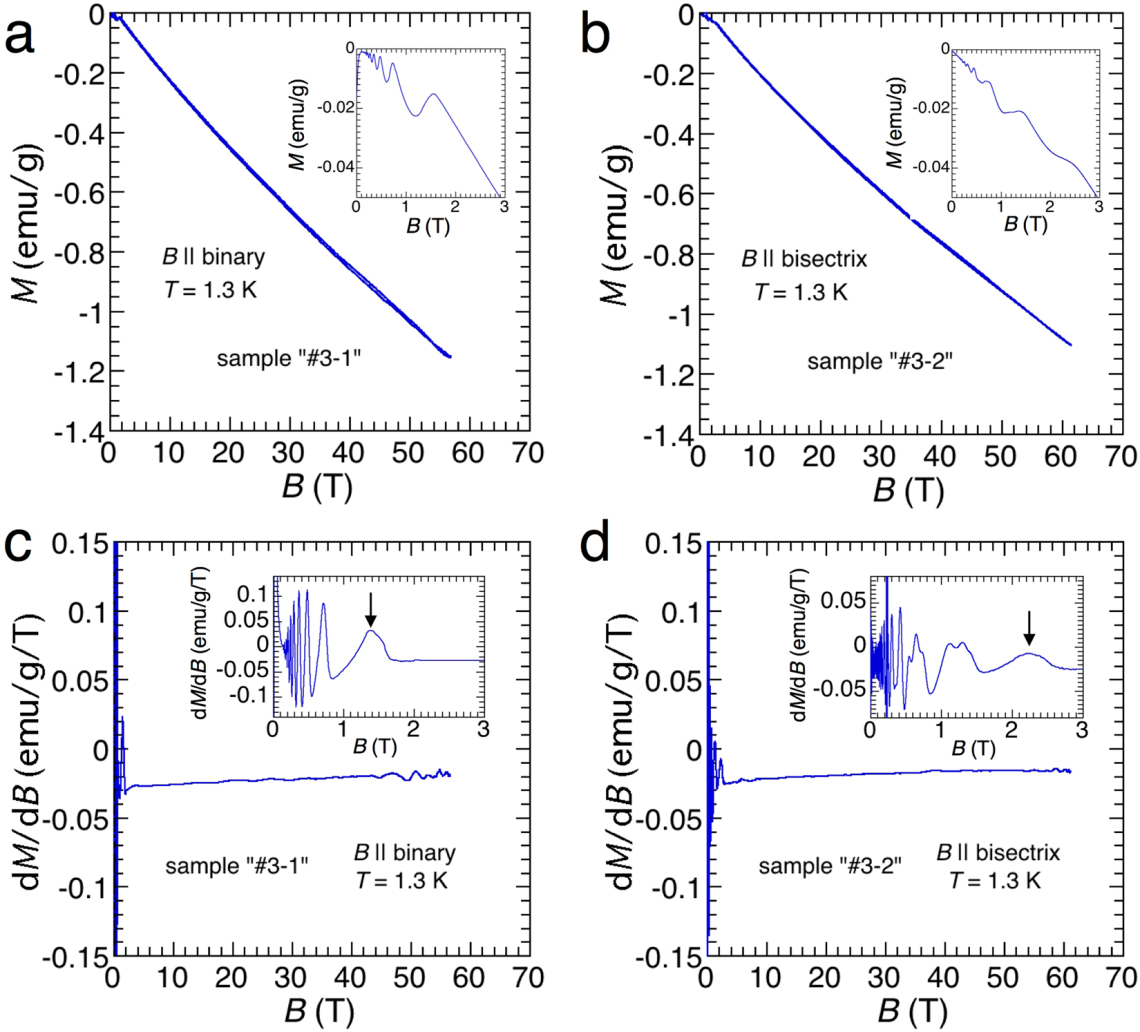
Magnetization of bismuth single crystals measured up to 60T for (**a**) *B* || binary and (**b**) *B* || bisectrix. First derivative of magnetization for (**c**) *B* || binary and (**d**) *B* || bisectrix in the field decreasing processes. The insets of (**a**–**d**) present magnified views of the main panel at the low-field range in the field decreasing processes. The arrows in the insets of (**c**,**d**) indicate the fields where the light electrons enter the quantum limit state.

To obtain thermodynamic information, we performed magnetization (*M*) measurements up to 60T. The samples for the magnetization measurements were taken from the same ingot used for the transport measurements. Figure 3 presents the observed magnetization curves at 1.3 K for *B* || binary (Fig. 3a) and *B* || bisectrix (Fig. 3b). In addition to the distinct de Haas–van Alphen (dHvA) oscillations in the low-field range shown in the insets, they show considerable diamagnetism exhibiting slight sub-linear behaviour as in the case of graphite^22^. The magnitude of diamagnetism at 60T is comparable with that in the Meissner state of a superconductor at a field of 14 mT. We also show the differential magnetization for *B* || binary (Fig. 3c) and *B* || bisectrix (Fig. 3d). The dHvA oscillations are clearly resolved in the low-field range, as shown in the insets of Fig. 3c,d. The observed dHvA oscillations in the low-field range are consistent with those obtained by the torque and Nernst measurements^23,24^. For *B* || binary, local maxima in the d*M*/d*B* curve below 1.4T can mainly be attributed to the LLs of the light electrons (*e*2 and *e*3)^23,24^. It can be expected that for both the previous torque and Nernst measurements, for *B* || binary, two light electron pockets (*e*2 and *e*3) and one heavy electron pocket (*e*1) will reach the quantum limit at about 1.4 and 10T, respectively^11^. For *B* || bisectrix, one light electron pocket (*e*1) and two heavy electron pockets (*e*2 and *e*3) will reach the quantum limit at about 1.2 and 2.3 T^11^. Our magnetization results clearly resolve the dHvA signals from the electron pockets at low fields except for those from the heavy electron pocket (*e*1) at approximately 10T for *B* || binary. In contrast, we cannot resolve the dHvA signals from the hole pockets above 10T. Earlier magnetic measurements indicated that the dHvA signals from the hole pockets were smaller than those from the electron pockets^23,25^. To resolve the quantum oscillations in the high-field range, we should measure the other physical quantities.

**Figure 4:**
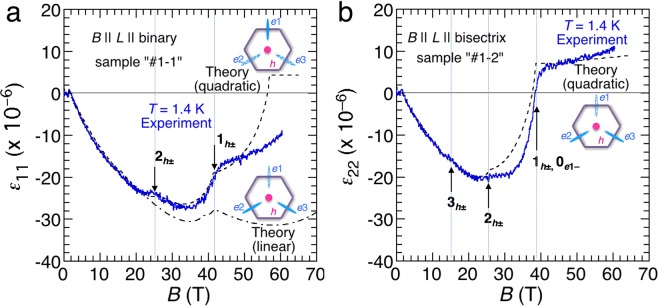
Field dependences of the longitudinal magnetostriction of bismuth single crystals together with the calculated curves. The experimental curves were obtained in pulsed magnetic fields up to 60T at 1.4 K for (**a**) *B* || binary and (**b**) *B* || bisectrix. The horizontal solid lines indicate the zero level of the magnetostriction. The vertical dotted lines and black arrows indicate the kinks of the experimental curves, which correspond to the Landau level (LL) crossing fields. The dashed (dash-dotted) line marked by quadratic (linear) is the theoretical curve of magnetostriction calculated by the quadratic (linear) model.

Thus, we measured the magnetostriction of bismuth. The blue solid lines in Fig. 4 represent the experimental results of the longitudinal magnetostriction at 1.4 K in pulsed magnetic fields up to 60T for *B* || binary (Fig. 4a) and *B* || bisectrix (Fig. 4b). The vertical axis represents the field-induced relative changes in the sample length (*ΔL/L*) along the binary (*ε*_11_) and bisectrix (*ε*_22_) axes, respectively. To eliminate any sample dependence, we used the same sample pieces as those used in the magnetotransport measurements (sample ‘#1-1’ and ‘#1-2’). For both field directions, the samples monotonically shrink up to 20T, consistent with the earlier reports^26,27^. The experimental curve for *B* || binary exhibits kinks at 26 and 42T, which correspond to the level crossings of 2_*h*±_ and 1_*h*±_ Landau subbands, respectively. Therefore, magnetostriction resolves the quantum oscillations from the hole pockets more sensitively compared to the magnetization. The result for *B* || bisectrix also presents small kinks at 16 and 27T, which correspond to the level crossings of 3_*h*±_ and 2_*h*±_ subbands, respectively, whereas there is a kink immediately above the significant sign reversal at approximately 39T, which corresponds to the level crossings of both 1_*h*±_ and 0_*e*1−_ subbands in the quadratic model.

**Figure 5:**
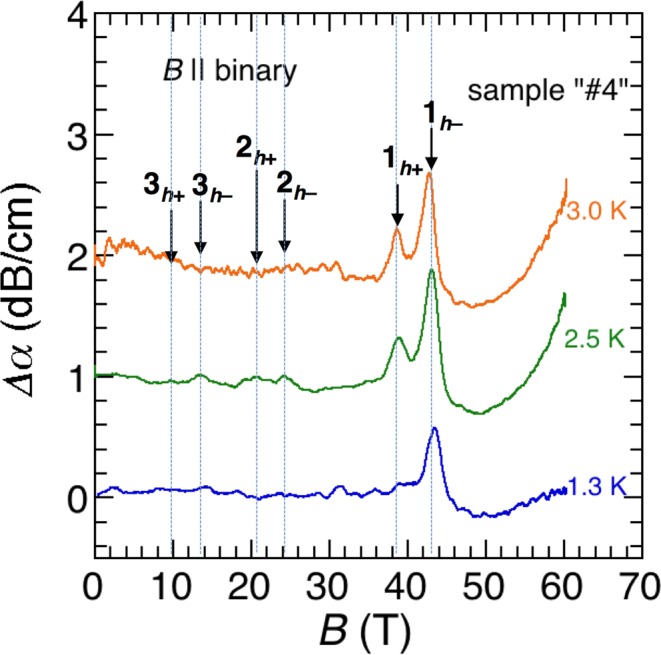
Ultrasound attenuation coefficient *Δα* of Bi single crystal for *B* || binary. Longitudinal acoustic waves were propagated along the binary axis at a frequency of *f* = 33 MHz. The vertical dotted lines correspond to the LL crossing fields guided by the quadratic model.

To more clearly resolve the quantum oscillations, we performed ultrasound measurements on bismuth. As shown in the earlier experiments, ultrasound attenuation can sensitively resolve the quantum oscillations in bismuth^28–30^. Figure 5 shows the ultrasound attenuation measured at 1.3, 2.5, and 3.0 K in pulsed magnetic fields up to 60T applied along the binary axis. As shown in Fig. 5, the ultrasound attenuation presents distinct peak structures at the hole LL crossing fields for small Landau indices. The sample used for the ultrasound measurements (sample ‘#4’) was taken from a different ingot than that used for the other measurements, but the peak fields reasonably coincide with those observed in the other experiments. Even in this highly sensitive experiment, there exists no distinct anomaly at approximately 50T, as observed in the second derivative of the MR shown in Fig. 2c. As indicated in Fig. 5, the ultrasound attenuation exhibits a steep increase in the field region above 50T, which implies the existence of an additional peak structure at fields higher than 60T.

## Discussion

First, we consider valley polarization in the high-field range based on the magnetostriction results. Magnetostriction is determined by the valance between the elastic energy and total energy of charge carriers in the occupied states, and thus provides direct information of the valley polarization. According to Michenaud *et al*., the magnetostriction of bismuth is associated with the densities of electrons by the following equations^26^:

1 $${\varepsilon }_{11}=-\,(\alpha +\beta ){\Delta }{N}_{e1}+(\alpha /2\,-\,\beta ){\Delta }{N}_{e2}+(\alpha /2\,-\,\beta ){\Delta }{N}_{e3}\,{\rm{for}}\,B\Vert {\rm{binary}}$$

2 $${\varepsilon }_{22}=(\alpha \,-\,\beta ){\Delta }{N}_{e1}\,-\,(\alpha /2+\beta ){\Delta }{N}_{e2}\,-\,(\alpha /2+\beta ){\Delta }{N}_{e3}\,{\rm{for}}\,B\Vert {\rm{bisectrix}}$$

here, *ΔN*_*e*1_, *ΔN*_*e*2_, and *ΔN*_*e*3_ denote field-induced changes of carrier densities in the three electron valleys (*e*1, *e*2, and *e*3, respectively). The coefficients *α* and *β* can be determined from the elastic compliance and deformation potential. As claimed by Michenaud *et al*., the estimation of *α* and *β* based on the early experimental data of compliance results in a slightly larger magnetostriction than the experimental results^26^. Therefore, we re-evaluated these coefficients with using the experimental results of *ε*_11_ and *ε*_22_ and theoretically calculated values of *ΔN*_*e*1_, *ΔN*_*e*2_, and *ΔN*_*e*3_ as a function of *B*. Using the theoretical carrier density values given in ref.^10^, we determined *α* = −2.17 × 10^−23^ cm^3^ and *β* = 6.97 × 10^−24^ cm^3^. These values are slightly different from the values previously reported (*α* = −1.905 × 10^−23^ cm^3^ and *β* = 8.64 × 10^−24^ cm^3^)^20^. The calculated magnetostriction data based on the quadratic model are represented by black dashed lines in Fig. 4a,b. The dash-dotted line in Fig. 4a represents the calculated data with using the carrier densities in the linear model^27^. As shown in these figures, the overall trend of experimental data is reproduced better by the quadratic model than by the linear model. In particular, the abrupt sign reversal in *ε*_22_ at approximately 39T for *B* || bisectrix is reasonably reproduced by the quadratic model. This sign reversal originates from the sign change in the first term (*ΔN*_*e*1_ < 0) at the right-hand side of equation (2), which reflects complete valley polarization. On the other hand, we do not observe sign reversal for *B* || binary up to 60T. This slight discrepancy may be solved by further fine tuning of the parameters used in the quadratic model.

Secondly, we discuss the origin of the anomaly at approximately 50T for *B* || binary observed only in the MR (Fig. 2a,c). According to the quadratic model, the LL crossings of both the 0_*e*2*−*_ and 0_*e*3*−*_ levels take place at approximately 57T for *B* || binary^10,11^. Since the onset fields of this LL crossing move to higher fields by rotating the field direction in the binary-bisectrix plane^10,11^, the observed anomaly cannot be simply ascribed to this LL crossing caused by a misalignment of the field. The results of the magnetostriction and ultrasound measurements imply that the lowest LLs of the light electrons (0_*e*2−_ and 0_*e*3−_) will be away from the Fermi energy; in other words, the complete polarization of the electron valleys will be realized above 60T. As expected by equation (1), a drastic sign reversal will be observed at the field where the light electrons depopulate. The anomaly observed in MR at approximately 50T is strongly damped and moves towards a slightly higher field with increasing temperature, unlike the other peaks. Therefore, we ascribe this anomaly to a different origin from the others caused by SdH oscillations. In addition, our results of SdH oscillations and magnetostriction almost coincide with those observed in refs^10,26^. Therefore, we do not consider this anomaly as that from the secondary crystallographic domains caused by twinning^31^.

**Figure 6:**
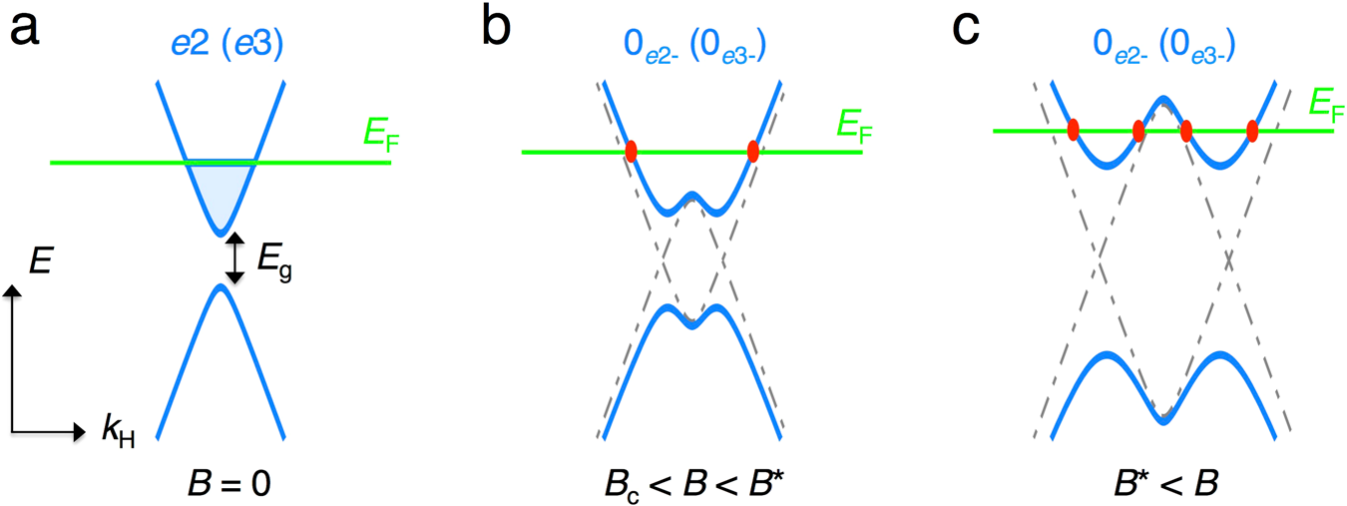
(**a**) Schematic of dispersion relation of an electron band *e*2 (or *e*3) at around the *L* point in zero field. (**b**,**c**) Expected dispersion relations of the lowest Landau subband in high magnetic fields applied along the binary direction (blue solid lines). The *k*_*H*_ represents the wave vector along the field direction. Here, B^*^ denotes the field at which local maximum of the dispersion cross the Fermi energy *E*_F_ (green solid lines). In this scenario, Fermi pocket of 0_*e*2−_ (0_*e*3−_) subband splits into two valleys at *B* > B^*^ as shown in (**c**).

We infer the origin of this anomaly as a field-induced splitting of the electron valleys. As mentioned before, the application of a magnetic field for *B* || binary is regarded to reduce the gap between the conduction and valence bands at the *L*-points illustrated in Fig. 6a. The finite interaction between these two bands realizes avoided level crossing at a certain field *B*_c_, as schematically shown in Fig. 6b. Here, the horizontal axis represents the wavenumber along the field direction (*k*_*H*_). In this case, the dispersion relation will deform to a ‘camel’s back’ structure along the *k*_*H*_ direction. As the field increases, Fermi energy (*E*_F_) will pass through the local maxima in the 0_*e*2−_ and 0_*e*3−_ subbands at a field of B^*^ prior to the complete depopulation of these subbands (Fig. 6c). During this process, each electron valley splits into to two valleys. This change in the FS topology is a kind of Lifshitz transition^32^, but is different from the magnetic breakdown that takes place in the reciprocal plane normal to the magnetic field^33–35^. Actually, the possible realization of the ‘camel’s back’ structures of the lowest LLs of the light electron valleys (0_*e*2−_ and 0_*e*3−_) for *B* || binary has been proposed in ref.^36^. In addition, the field-induced change in the SdH frequency suggests that the topological change in the FSs is observed in antimony-doped bismuth under uniaxial compression^34,35,37^. As the two models used in our analysis do not consider the effect of *k*_*H*_-dispersion as *k*_*H*_ = 0, the emergence of this additional anomaly cannot be anticipated in these models.

At the moment, it is not clear why we do not observe this anomaly in the physical quantities other than the MR. Contrary to thermodynamic measurements, anomalies in MR are comparatively magnified through the large factor of (*ω*_c_*τ*)^2^, where *ω*_c_ and *τ* denote the cyclotron frequency and the relaxation time^38^. In recent experiments of heavy fermion systems, similar kinds of changes in topology of the FSs manifest as distinct anomalies in the transport properties, whereas there are no distinct anomalies in the thermodynamic quantities^39–42^. In addition, it is also not clear why we do not observe similar anomaly for *B* || bisectrix. The depopulation field for the 0_*e*1−_ valley (~40T) might be too small to realize the pronounced camel’s back structure detectable in experiments. Moreover, MR for *B* || binary is more sensitive to the valley polarization than that for *B* || bisectrix because of the anisotropy in the mobility, as pointed out in ref.^10^. This may result in emergence of this anomaly only for *B* || binary. Although we propose topological change in FS as a possible origin for this anomaly, we do not rule out the other scenario. Further experimental studies are needed to clarify what is going on at around 50T for *B* || binary.

In summary, we studied various physical properties of bismuth single crystals in magnetic fields up to 60T. The observed quantum oscillations are reasonably explained by a theoretical model that includes a quadratic *B* dependence term in the Zeeman energy, except for an anomaly observed in the MR at approximately 50T for *B* || binary. We proposed field-induced splitting of the electron valleys as a possible origin for this anomaly. In contrast, our magnetostriction results for *B* || bisectrix present a sign reversal at approximately 39T, suggesting the complete depopulation of one of the three electron valleys at this field, which is also reasonably explained by the theoretical model.

## Methods

### Sample preparation

Sample specimens were spark-cut from the ingot of bismuth single crystals grown by the Czochralski method, and these surfaces were then etched in dilute nitric acid (HNO_3_:H_2_O = 3:7) for about 3–5 min. The principal axes of the crystals were determined within an accuracy of ±0.5° by the back-reflection Laue method. The ratio of room-temperature to residual resistivity was in the range of 80–200. Typical dimensions of the samples were 2 × 2 × 3 mm^3^ for ultrasonic attenuation, 3 × 3 × 4-9 mm^3^ for MR and magnetostriction, and 2 × 2 × 9 mm^3^ for magnetization measurements. Samples ‘#1-1’, ‘#1-2’, ‘#2-1’, and ‘#2-2’ were used for the MR measurements, ‘#1-1’ and ‘#1-2’ were also used for the magnetostriction measurements, ‘#3-1’ and ‘#3-2’ were used for the magnetization measurements, and ‘#4’ was used for the ultrasonic attenuation measurement. The samples used for the MR, magnetization, and magnetostriction measurements were taken from the same ingot.

### Measurement apparatus

MR measurements were performed in the transverse MR configuration with a standard four-probe technique. The resistance was measured using 100-kHz AC current and analysed using a custom low-noise digital lock-in technique. Electrical current was applied along the trigonal direction. Copper wires of 60 μm in diameter were attached to four electrodes on the sample using Wood’s metal. Magnetization measurements were performed using standard induction methods. Magnetostriction measurements were performed in the longitudinal magnetostriction configuration by the capacitance method using a capacitance bridge (1615-A, IET LABS, Inc.). Ultrasound measurements were performed with the pulse-echo method at a fixed frequency of 33 MHz. A pair of LiNiO_3_ single crystals with a thickness of about 0.1 mm was used as ultrasound transducers. All the high-fields experiments were performed in pulsed magnetic fields using a solenoid-type magnet that can produce 60T with a pulse duration of about 4 ms (for magnetization measurements) or 36 ms (for the other measurements) at the International MegaGauss Science Laboratory at ISSP of the University of Tokyo.

## References

[1] Xiao D, Yao W, Niu Q (2007). Valley-contrasting physics in graphene: Magnetic moment and topological transport. Phys. Rev. Lett..

[2] Behnia K (2012). Polarized light boosts valleytronics. Nat. Nanotech..

[3] Nebel CE (2013). Electrons dance in diamond. Nat. Mater..

[4] Zeng H, Dai J, Yao W, Xiao D, Cui X (2012). Valley polarization in MoS_2_ monolayers by optical pumping. Nat. Nanotechnol..

[5] Isberg J (2013). Generation, transport and detection of valley-polarized electrons in diamond. Nat. Mater..

[6] Zhu Z, Collaudin A, Fauqué B, Kang W, Behnia K (2012). Field-induced polarization of Dirac valleys in bismuth. Nat. Phys..

[7] Jo YJ (2014). Valley-polarized interlayer conduction of anisotropic Dirac Fermions in SrMnBi_2_. Phys. Rev. Lett..

[8] Collaudin A, Fauqué B, Fuseya Y, Kang W, Behnia K (2015). Angle dependence of the orbital magnetoresistance in bismuth. Phys. Rev. X.

[9] Kumar N, Shekhar C, Klotz J, Wosnitza J, Felser C (2017). Magnetic field induced strong valley polarization in the three-dimensional topological semimetal LaBi. Phys. Rev..

[10] Zhu Z (2017). Emptying Dirac valleys in bismuth using high magnetic fields. Nat. Commun..

[11] Zhu Z, Fauqué B, Behnia K, Fuseya Y (2018). Magnetoresitance and valley degree of freedom in bulk bismuth. J. Phys. Condens. Mater.

[12] Fuseya Y, Ogata M, Fukuyama H (2015). Transport properties and diamagnetism of Dirac electrons in bismuth. J. Phys. Soc. Jpn..

[13] Küchler R (2014). Thermodynamic evidence for valley-dependent density of states in bulk bismuth. Nat. Mater..

[14] Wolff PA (1964). Matrix elements and selection rules for the two-band model of bismuth. J. Phys. Chem. Solids.

[15] Fukuyama H, Kubo R (1970). Interband effects on magnetic susceptibility. II. Diamagnetism of bismuth. J. Phys. Soc. Jpn..

[16] Hiruma K, Miura N (1983). Magnetoresistance study of Bi and Bi–Sb alloys in high magnetic fields. II. Landau levels and semimetal-semiconductor transition. J. Phys. Soc. Jpn..

[17] Baraff GA (1965). Magnetic energy levels in the bismuth conduction band. Phys. Rev..

[18] Vecchi MP, Pereira JR, Dresselhaus MS (1976). Anomalies in the magnetoreflection spectrum of bismuth in the low-quantum-number limit. Phys. Rev. B.

[19] Miura N, Hiruma K, Kido G, Chikazumi S (1982). Observation of the magnetic-field-induced semimetal-semiconductor transition in Bi under megagauss fields. Phys. Rev. Lett..

[20] Heremans J, Michenaud J-P (1985). Electronic magnetostriction of Bi_1−*x*_Sb_*x*_ alloys. J. Phys. C: Solid State Phys..

[21] Miura N, Clark RG, Newbury R, Starrett RR, Skougarevsky AV (1994). Anomalous magnetoresistance and Shubnikov-de Haas effect observed for bismuth in pulsed magnetic fields up to 50T at temperatures down to 0.3K. Physica B.

[22] Akiba K (2015). Possible excitonic phase of graphite in the quantum limit state. J. Phys. Soc. Jpn..

[23] Saito Y (1963). Spin splitting of the Landau levels in bismuth observed by the de Haas–van Alphen effect. J. Phys. Soc. Jpn..

[24] Zhu Z, Fauqué B, Fuseya Y, Behnia K (2011). Angle-resolved Landau spectrum of electrons and holes in bismuth. Phys. Rev. B.

[25] Brandt NB, Dubrovskaya AE, Kytin GA (1960). An investigation of the quantized oscillations in the magnetic susceptibility of bismuth at extremely low temperatures. *Soviet Phys*. JETP.

[26] Michenaud J-P, Heremans J, Shayegan M, Haumont C (1982). Magnetostriction of bismuth in quantizing magnetic fields. Phys. Rev. B.

[27] Iye Y (1983). Magnetostriction of bismuth and graphite in fields up to 40 Tesla. J. Phys. Soc. Jpn..

[28] Mase S, Sakai T (1971). A new phenomenon is sound attenuation in bismuth under very strong magnetic fields. J. Phys. Soc. Jpn..

[29] Sakai T, Goto N, Mase S (1973). Further study of the occurrence of the excitonic phase transition in bismuth under strong magnetic fields by ultrasonic attenuation. J. Phys. Soc. Jpn..

[30] Kajimura K, Tokumoto H, Inaba R, Mikoshiba N (1975). Anomalous behavior of giant quantum attenuation in bismuth. Phys. Rev. B.

[31] Zhu Z (2012). Landau spectrum and twin boundaries of bismuth in the extreme quantum limit. PNAS.

[32] Lifshitz IM (1960). Anomalies of electron characteristics of a metal in the high pressure region. Sov. Phys. JETP.

[33] Guthmann C, Thuillier JM (1970). Fermi surface of tellurium. Phys. Stat. Sol. (b).

[34] Lavrenyuk MY, Minina NY, Savin AM (1987). Formation of a dumbbell Fermi surface and intraband magnetic breakdown due to uniaxial compression of the alloy Bi_0.78_Sb_0.22_. JETP Lett..

[35] Minina NY, Lavrenyouk MY, Savin AM (1991). Saddle point in energy spectrum and intraband magnetic breakdown in semiconducting bismuth-antimony alloys under strong uniaxial pressure. Semicond. Sci. Technol..

[36] Vecchi, M. P., Pereira, J. R. & Dresselhaus, M. S. Magnetic field-induced semimetal-semiconductor transition in Bi. *Proc*. *Int*. *Conf*. *Phys*. *Semicond*. (ed. Teubner, B. G.) 1181–1185 (Stuttgart, 1974).

[37] Varlamov AA, Egorov VS, Pantsulaya AV (1989). Kinetic properties of metals near electronic topological transitions (2 1/2-order transitions). Adv. Phys..

[38] Blanter YM, Kaganov MI, Pantsulaya AV, Varlamov AA (1994). The theory of electronic topological transitions. Phys. Rep..

[39] Pfau H (2013). Interplay between Kondo suppression and Lifshitz transitions in YbRh_2_Si_2_ at high magnetic fields. Phys. Rev. Lett..

[40] Aoki D (2016). Field-induced Lifshitz transition without metamagnetism in CeIrIn_5_. Phys. Rev. Lett..

[41] Bastien G (2016). Lifshitz transitions in the ferromagnetic superconductor UCoGe. Phys. Rev. Lett..

[42] Pfau H (2017). Cascade of magnetic-field-induced Lifshitz transitions in the ferromagnetic Kondo lattice material YbNi_4_P_2_. Phys. Rev. Lett..

